# Novel Synthesis of Phosphorus-Doped Porous Carbons from Lotus Petiole Using Sodium Phytate for Selective CO_2_ Capture

**DOI:** 10.3390/molecules30193990

**Published:** 2025-10-05

**Authors:** Yue Zhi, Jiawei Shao, Junting Wang, Xiaohan Liu, Qiang Xiao, Muslum Demir, Utku Bulut Simsek, Linlin Wang, Xin Hu

**Affiliations:** 1 Key Laboratory of the Ministry of Education for Advanced Catalysis Materials, Zhejiang Normal University, Jinhua 321004, China; 15034243438@163.com (Y.Z.); shaojw2001@outlook.com (J.S.); wwwjting@163.com (J.W.); 19548990836@163.com (X.L.); 2 Institute of Plant Nutrition, Resources and Environment, Beijing Academy of Agriculture and Forestry Science, Beijing 100097, China; xqiang1978@163.com; 3 Department of Chemical Engineering, Bogazici University, Istanbul 34342, Türkiye; demirm@alumni.vcu.edu; 4 TUBITAK Marmara Research Center, Material Institute, Gebze 41470, Türkiye; 5 Department of Machine, Anadolu BIL Vocational School, Istanbul Aydin University, Istanbul 34295, Türkiye; utkubulutsimsek@aydin.edu.tr

**Keywords:** phosphorus-doped porous carbon, CO_2_ adsorption, sodium phytate activation, biomass-derived carbon

## Abstract

Developing sustainable and high-performance sorbents for efficient CO_2_ capture is essential for mitigating climate change and reducing industrial emissions. In this study, phosphorus-doped porous carbons (LPSP-T) were synthesized via a one-step activation–doping strategy using lotus petiole biomass as a precursor and sodium phytate as a dual-function activating and phosphorus-doping agent. The simultaneous activation and phosphorus incorporation at various temperatures (650–850 °C) under a nitrogen atmosphere produced carbons with tailored textural properties and surface functionalities. Among them, LPSP-700 exhibited the highest specific surface area (525 m^2^/g) and a hierarchical porous structure, with abundant narrow micropores (<1 nm) and phosphorus-containing surface groups that synergistically enhanced CO_2_ capture performance. The introduction of P functionalities not only improved the surface polarity and binding affinity toward CO_2_ but also promoted the formation of a well-connected pore network. As a result, LPSP-700 delivered a CO_2_ uptake of 2.51 mmol/g at 25 °C and 1 bar (3.34 mmol/g at 0 °C), along with a high CO_2_/N_2_ selectivity, fast CO_2_ adsorption kinetics and moderate isosteric heat of adsorption (*Q_st_*). Furthermore, the dynamic CO_2_ adsorption capacity (0.81 mmol/g) was validated by breakthrough experiments, and cyclic adsorption–desorption tests revealed excellent stability with negligible loss in performance over five cycles. Correlation analysis revealed pores < 2.02 nm as the dominant contributors to CO_2_ uptake. Overall, this work highlights sodium phytate as an effective dual-role agent for simultaneous activation and phosphorus doping and validates LPSP-700 as a sustainable and high-performance sorbent for CO_2_ capture under post-combustion conditions.

## 1. Introduction

The uncontrolled rise in atmospheric CO_2_ concentrations, primarily due to fossil fuel combustion, poses a serious threat to global climate stability. Among all greenhouse gases, CO_2_ accounts for the highest percentage of human-induced emissions and has a critical role in global warming and ocean acidification [1]. Consequently, the development of efficient, cost-effective, and environmentally benign technologies for CO_2_ capture and storage (CCS) has become a global research priority [2]. Traditional CCS technologies, e.g., solvent-based amine scrubbing [3], though efficient, bear drawbacks such as high energy penalty, corrosion, and secondary pollution. In contrast, adsorption using solid sorbents offers multiple advantages, including lower regeneration energy demand, enhanced operational stability, tunable surface chemistry, and reduced risk of corrosion [4]. Moreover, solid sorbents are generally easier to handle, can be regenerated under milder conditions, and produce minimal secondary environmental impact, making them attractive candidates for sustainable CO_2_ capture. 

A wide range of solid sorbents have been explored for this purpose, such as zeolites [5], metal–organic frameworks (MOFs) [6,7], porous carbons [8,9,10,11,12,13,14,15], and porous polymers [16,17,18]. Among these, porous carbons stand out owing to their high surface areas, adjustable pore structures, and excellent chemical robustness, while also allowing heteroatom doping to further improve CO_2_ affinity [19,20,21,22]. These characteristics make porous carbons one of the most promising classes of sorbents for practical applications. Within this category, renewable biomass-derived carbons are particularly appealing for CCS applications because of their low cost, sustainability, and structural versatility [23,24]. Biomass feedstocks, such as agricultural residues, aquatic plants, and lignocellulosic wastes, can be converted into porous carbonaceous materials with well-developed porosity and tailored surface chemistry through thermal activation processes [25]. In this context, lotus petiole, an abundant and underutilized agricultural byproduct, represents a promising precursor due to its natural porosity, high oxygenated functional content, and renewable origin, thereby providing an environmentally benign and sustainable route for the synthesis of functional porous carbons.

Heteroatom doping has been demonstrated as an effective strategy to enhance the CO_2_ capture performance of porous carbons. While nitrogen and sulfur doping have been extensively studied [26,27,28,29,30,31,32,33], research on phosphorus doping remains relatively limited, despite its unique ability to introduce polar surface functional groups (e.g., P-O and P-O) and modulate the electronic properties of carbon frameworks [34,35]. These P-containing functionalities can amplify dipole–quadrupole interactions and improve Lewis acid–base interactions with CO_2_ molecules, thereby enhancing adsorption affinity, especially at low partial pressures [36,37]. Furthermore, phosphorus doping often promotes the formation of hierarchical pore structures during activation, which benefits diffusion kinetics and gas accessibility [38].

Various phosphorus sources, such as phosphoric acid [39], ammonium phosphate [40], and triphenylphosphine [41], have been employed for carbon activation and doping; however, these reagents often suffer from environmental concerns, high costs, or limited efficiency. Sodium phytate, a naturally occurring, phosphorus-rich compound composed of inositol hexaphosphate, represents a promising alternative. Upon thermal treatment, sodium phytate can decompose to generate P-containing functional groups while simultaneously facilitating pore creation. Nevertheless, its application in the activation of biomass-derived carbons for CO_2_ capture remains largely unexplored.

In this work, we address this gap by employing lotus petiole as a renewable precursor and sodium phytate as both activating and phosphorus-doping agent to synthesize porous carbons. To the best of our knowledge, this study represents the first comprehensive investigation integrating lotus petiole with sodium phytate for the fabrication of phosphorus-doped porous carbons and systematic evaluation of their CO_2_ capture performance. This strategy not only advances the sustainable production of functional porous carbons but also broadens the scope of phosphorus doping in carbon capture research.

In this work, we report a one-step synthesis method to phosphorus-doped porous carbons using lotus petiole, a low-cost, renewable biomass precursor, and sodium phytate as a dual doping and activation agent. The activation was conducted at various temperatures (650–850 °C) in nitrogen atmosphere, allowing for the fine-tuning of their textural and chemical properties. The effects of activation temperature on surface area, pore size distribution, elemental composition, and CO_2_ adsorption behavior were systematically explored. Additionally, the CO_2_ adsorption capacities of the porous boron-doped materials were evaluated under conditions of 0 °C and 25 °C at 1 bar pressure. The carbon dioxide capture performance of the materials was comprehensively investigated by analyzing their textural and surface properties in detail.

## 2. Synthesis and Characterization

The lotus petiole biomass was initially washed with deionized water to eliminate dust and surface contamination. The purified material was then oven-dried at 105°C for 24 h and then ground and sieved to a particle size between 100 and 140 mesh (105–150 μm). The dry powder was placed in a horizontal quartz tube furnace and carbonized at continuous flow of nitrogen (200 mL/min). The furnace temperature was increased from room temperature to 500 °C at a heating rate of 5 °C/min and kept for 2 h. On simultaneously cooling in nitrogen, the material thus obtained was named as LPC. An accurate amount of LPC was mixed with sodium phytate in a 1:2 weight ratio. The homogeneous mixture was charged into a tubular furnace and treated at different temperatures (650, 700, 750, 800, and 850 °C) for 2 h under nitrogen flow (400 mL/min). The ramping rate was 5 °C/min. The resulting black powder was cleaned with hot deionized water a few times to remove the remaining salt and dried overnight in 80 °C. The resulting products were designated as LPSP-T, where T is the activation temperature. The schematic synthesis process for these P-doped porous carbons can be found in Figure S1 of the Supplementary Materials. Comprehensive details regarding the synthesis process, characterization of the adsorbents, and the measurement of CO_2_ adsorption performance are provided in the Supplementary Materials.

## 3. Results and Discussion

### 3.1. Characterization Results of Porous Carbon

**Figure 1:**
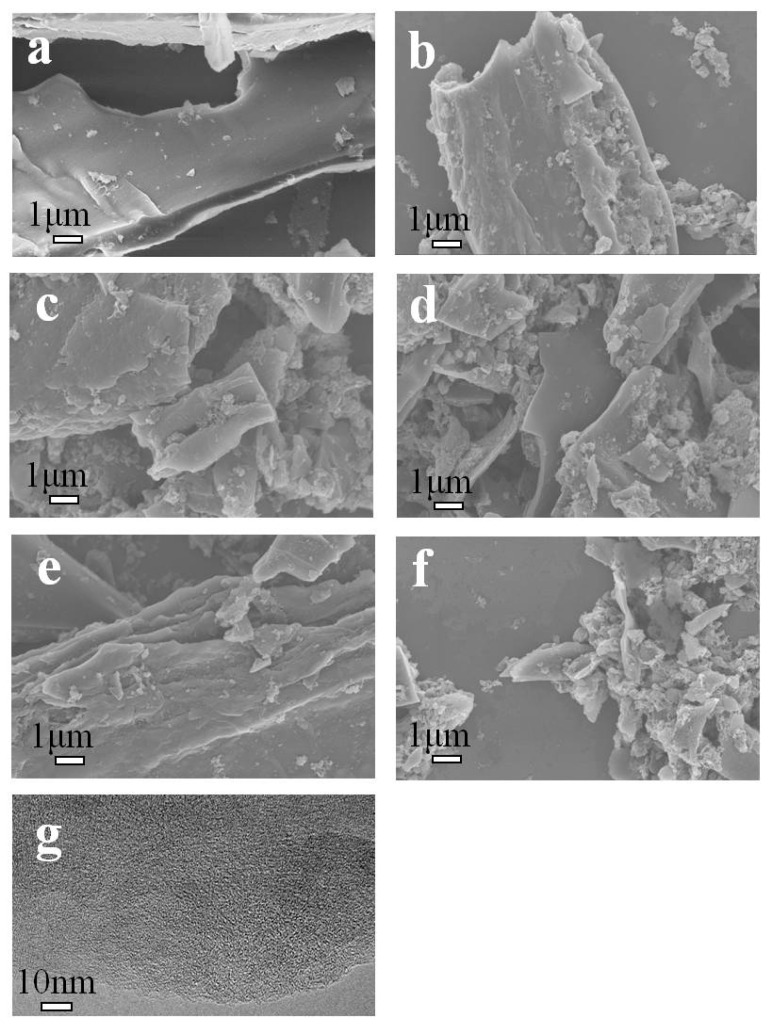
SEM of (**a**) LPC, (**b**) LPSP-650, (**c**) LPSP-700, (**d**) LPSP-750, (**e**) LPSP-800, (**f**) LPSP-850 and TEM of (**g**) LPSP-700.

The morphology of the as-prepared phosphorus-doped porous carbons (LPSP-T) was systematically investigated using scanning electron microscopy (SEM, Hitachi S-4800, Hitachi, Tokyo, Japan) and transmission electron microscopy (TEM, JEOL-2100F, JEOL, Kyoto, Japan) to assess the evolution of the carbon structure with varying activation temperatures. The SEM images (Figure 1a–f) show that the pristine carbonized lotus petiole (LPC) possessed a relatively smooth and compact surface with limited porosity. Upon activation with sodium phytate at elevated temperatures, the samples exhibited progressively rougher surface morphologies, accompanied by the emergence of abundant micro- and mesopores. This structural transformation can be attributed to the chemical activation process, in which the decomposition of sodium phytate etches the carbon matrix and generates hierarchical pores. The TEM image of LPSP-700 (Figure 1g) further confirmed the presence of a well-developed porous network, characterized by a disordered, amorphous carbon matrix interspersed with nano-sized pores. The combination of micro- and mesopores is particularly advantageous for gas adsorption, as micropores provide abundant active sites for CO_2_ capture, while mesopores enhance diffusion kinetics, ensuring rapid mass transport throughout the material. Collectively, these morphological features are highly favorable for CO_2_ adsorption, as they increase the accessibility of adsorption sites, strengthen gas–solid interactions, and contribute to the observed high CO_2_ uptake capacity.

**Figure 2:**
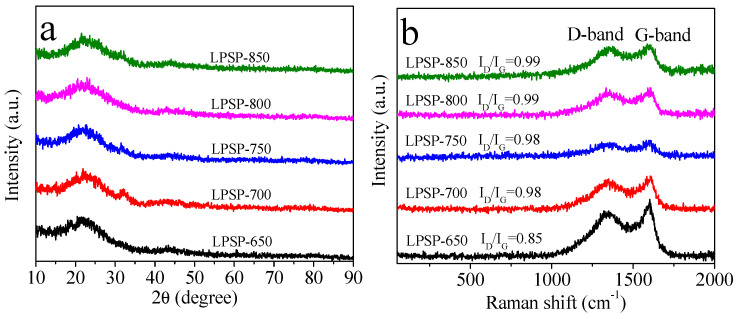
(**a**) XRD patterns and (**b**) Raman spectra of P-doped porous carbons.

The crystalline nature of the LPSP-T materials was investigated using X-ray diffraction (XRD). As shown in Figure 2a, all samples display two broad diffraction peaks at 2θ ≈ 23° and 43°, corresponding to the (002) and (100) planes of carbon, respectively. The broadness and low intensity of these peaks indicate a predominantly amorphous carbon structure with limited graphitic ordering, which is typical for heteroatom-doped carbons derived from biomass. This amorphous nature is likely beneficial for CO_2_ adsorption, as it can provide a higher density of edge sites and structural defects that serve as active adsorption centers. Complementary structural insights were obtained through Raman spectroscopy (Figure 2b). The spectra displayed two prominent bands: the D-band at approximately 1345 cm^−1^, associated with disordered carbon or defects, and the G-band at approximately 1590 cm^−1^, corresponding to in-plane vibrations of sp^2^-bonded carbon atoms. The intensity ratio of these bands (I_D_/I_G_) serves as an indicator of structural disorder. For LPSP-T samples activated at temperatures above 650 °C, the I_D_/I_G_ ratio ranged from 0.98 to 0.99, confirming a high defect concentration within the carbon framework. Such structural disorder is advantageous for gas adsorption because defects and heteroatom incorporation create additional active sites and enhance polar interactions with CO_2_ molecules. Moreover, the combination of amorphous structure and abundant defects can facilitate gas penetration and diffusion, thereby improving adsorption kinetics and overall uptake capacity.

**Figure 3:**
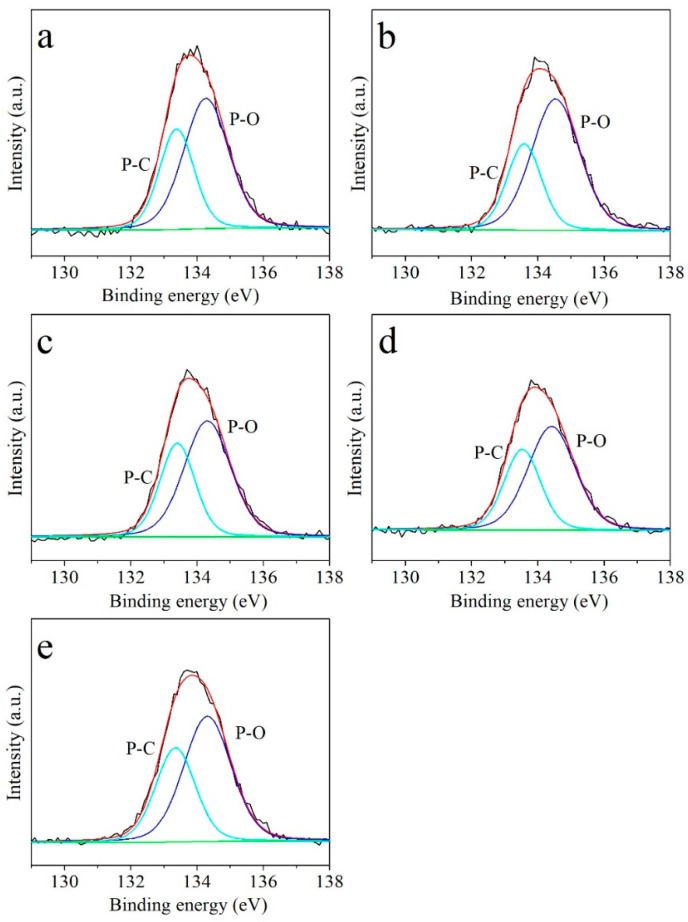
XPS P2p spectrum of (**a**) LPSP-650, (**b**) LPSP-700, (**c**) LPSP-750, (**d**) LPSP-800 and (**e**) LPSP-850.

X-ray photoelectron spectroscopy (XPS) was employed to investigate the surface elemental composition and chemical bonding of the LPSP-T samples. Survey spectra confirmed the presence of carbon (C), oxygen (O), phosphorus (P), and trace amounts of nitrogen (N) in all samples, indicating successful heteroatom incorporation. As summarized in Table 1, the phosphorus content reached a maximum of 3.28 at.% in LPSP-750, while LPSP-700 also exhibited a high P concentration of 2.92 at.%. The observed O and P contents primarily originate from the thermal decomposition of sodium phytate, which served both as a phosphorus dopant and a chemical activating agent during carbonization. High-resolution P 2p spectra (Figure 3a–e) provided more detailed insights into the chemical state of phosphorus. Deconvolution of the P 2p peaks revealed two dominant components at binding energies of 133.4 eV and 134.4 eV, corresponding to P–C and P–O bonds, respectively. These results confirm the successful incorporation of phosphorus species into the carbon framework. Notably, the P–O functionalities introduce polar surface sites, enhancing the sorbent’s surface polarity and promoting stronger interactions with CO_2_ molecules through Lewis acid–base and dipole–quadrupole interactions [38]. Such surface modifications are critical for improving CO_2_ adsorption capacity, especially at low partial pressures, by providing additional active sites and increasing the affinity of the carbon matrix toward acidic gas molecules. Moreover, the uniform dispersion of phosphorus species throughout the carbon matrix may also contribute to the creation of hierarchical porosity, facilitating gas diffusion and enhancing overall adsorption kinetics.

### 3.2. Textural Properties

The textural properties of the LPSP-T phosphorus-doped porous carbons were thoroughly characterized to establish the relationship between pore structure and CO_2_ adsorption performance. Key parameters, including specific surface area (S_BET_), total pore volume (V_0_), microporous pore volume (V_t_), and pore size distribution, were determined via nitrogen (N_2_) physisorption analyses. In addition, the volumes of narrow micropore (<1 nm) (V_n_) were obtained through Dubinin-Radushkevich (D-R) equation using CO_2_ adsorption data at 0 °C, which are known to critically influence CO_2_ sorption behavior under low pressure.

**Figure 4:**
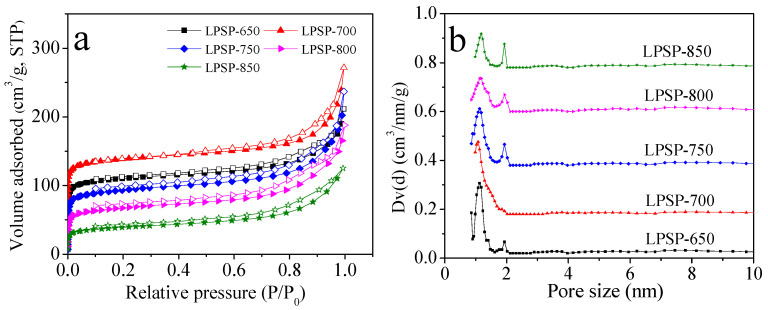
(**a**) N_2_ sorption isotherms and (**b**) pore size distribution of the samples prepared at different conditions. Filled and empty symbols in (**a**) represent adsorption and desorption branches, respectively.

The N_2_ adsorption–desorption isotherms recorded at –196 °C (Figure 4a) exhibited mixed characteristics of type I and type IV, indicative of the coexistence of micropores and mesopores. A steep increase in adsorption volume at low relative pressures (P/P_0_ < 0.1) reflects a high degree of microporosity, whereas the pronounced hysteresis loop at higher relative pressures (P/P_0_ > 0.4) reflects capillary condensation within mesopores [42,43]. Among the series, LPSP-700 demonstrated the highest BET surface area (525 m^2^/g) and total pore volume (0.39 cm^3^/g), highlighting its superior textural development. In comparison, LPSP-650 and LPSP-750 exhibited moderate surface areas of 418 m^2^/g and 341 m^2^/g, respectively, while LPSP-800 and LPSP-850 showed a significant decline (237 m^2^/g and 132 m^2^/g, respectively), which can be attributed to pore coalescence or collapse at elevated activation temperatures, limiting accessible surface area and pore connectivity [44]. Pore size distribution derived from NLDFT simulations of the N_2_ isotherms (Figure 4b) revealed that all samples were dominated by micropores with widths below 2 nm, which are particularly favorable for CO_2_ adsorption under sub-ambient and ambient conditions [45]. Quantitative analysis based on CO_2_ adsorption isotherms at 0 °C indicated that the narrow micropore volume (<1 nm) was highest for LPSP-700 and LPSP-800 (0.28 cm^3^/g), followed closely by LPSP-750 (0.26 cm^3^/g). Although LPSP-850 retained a comparable micropore volume, its reduced total pore volume and surface area suggested partial structural collapse at 850°C. LPSP-650, despite a slightly lower narrow micropore volume (0.25 cm^3^/g), still exhibited substantial CO_2_ capacity owing to its relatively high surface area.

Overall, these findings highlight that LPSP-700 possesses a well-balanced hierarchical pore structure, with an optimal combination of micro- and mesopores and a high narrow micropore fraction. This structural arrangement maximizes molecular sieving and confinement effects, facilitates efficient gas diffusion, and ensures high accessibility of adsorption sites, all of which are critical factors for selective and high-capacity CO_2_ capture. The analysis further demonstrates that careful tuning of activation temperature is essential for optimizing textural properties, and that a balance between narrow micropore volume and total pore connectivity is key to maximizing CO_2_ adsorption performance.

### 3.3. CO_2_ Adsorption Results of Porous Carbon

**Figure 5:**
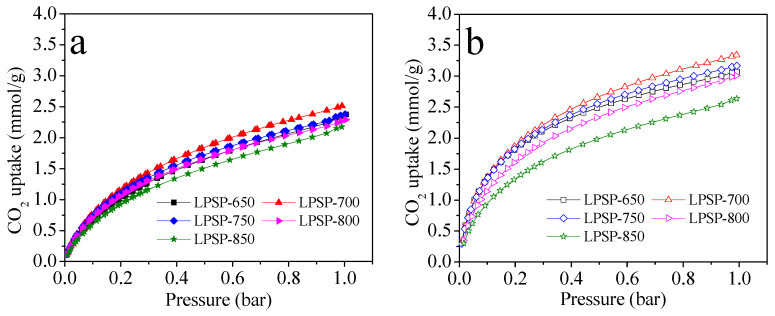
CO_2_ adsorption isotherms at (**a**) 25 °C and (**b**) 0 °C for P-doped LPSP porous carbons.

The CO_2_ adsorption isotherms of the phosphorus-doped LPSP porous carbons measured at 25 °C and 0 °C (Figure 5a,b) provide valuable insights into the interplay between textural features, surface chemistry, and gas adsorption performance. All samples exhibited a pronounced uptake at low pressures, indicative of a strong affinity between CO_2_ molecules and the adsorbent surface. Among the series, LPSP-700 demonstrated the highest CO_2_ adsorption capacity, reaching 2.51 mmol/g at 25 °C and 3.34 mmol/g at 0 °C under 1 bar, outperforming both lower- and higher-temperature activated samples. The adsorption performance of the lotus petiole–derived phosphorus-doped carbons is comparable to, or even surpasses, those obtained from non-sustainable phosphorus sources. For instance, cornstalk-derived carbons doped with melamine and phytic acid exhibited a CO_2_ uptake of approximately 3.11 mmol/g at 25 °C and 1 bar [46]. Similarly, sucrose-based carbons prepared with phosphoric acid using MCM-22 as a template achieved a maximum capacity of 1.43 mmol/g [37]. A resorcinol–formaldehyde porous carbon synthesized with phosphoric acid and F108 as a template delivered an uptake of 3.64 mmol/g [47], while carbons obtained through H_3_PO_4_-assisted polymerization showed an adsorption capacity of 2.59 mmol/g [48]. These comparisons demonstrate that the sustainable sorbent developed in this work provides competitive CO_2_ capture capacity relative to conventional, non-renewable phosphorus-based counterparts.

The superior performance of LPSP-700 can be attributed to its optimal combination of textural and chemical characteristics. Structurally, it possesses the highest BET surface area (525 m^2^/g) and largest total pore volume (0.39 cm^3^/g), along with a well-developed narrow micropore fraction (V_n_ = 0.28 cm^3^/g), collectively maximizing the availability of adsorption sites and enhancing molecular sieving effects. These micropores provide confinement for CO_2_ molecules, enhancing adsorption at low pressures, while the mesoporous network ensures efficient gas diffusion and rapid adsorption kinetics.

In addition to favorable textural features, surface chemistry plays a critical role in adsorption performance. XPS analysis shows significant heteroatom incorporation in LPSP-700, with 1.58 at.% N, 2.92 at.% P, and 22.44 at.% O. These heteroatoms introduce basic and polar functional sites, enhancing the interaction between CO_2_ molecules and the carbon surface via Lewis acid–base interactions and quadrupole–dipole forces [34,35,36]. In particular, phosphorus doping introduces polar P–O and P–C functionalities, which not only increase surface polarity but also synergistically interact with nitrogen and oxygen species to strengthen CO_2_ binding, especially under ambient conditions. 

**Figure 6:**
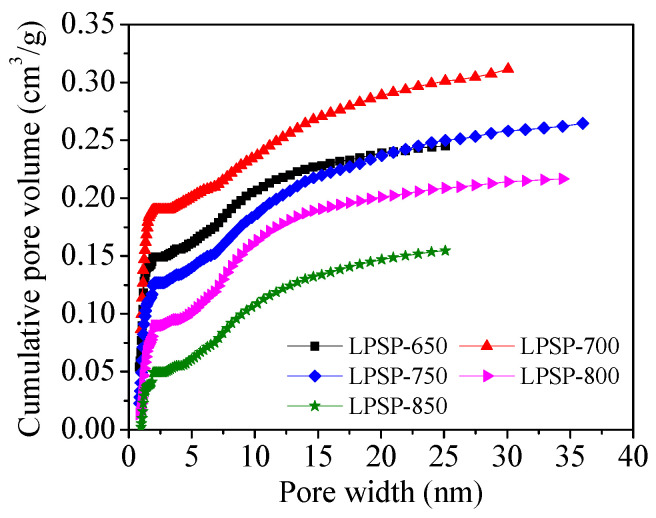
Cumulative pore volume as a function of pore width for LPSP-T samples.

**Figure 7:**
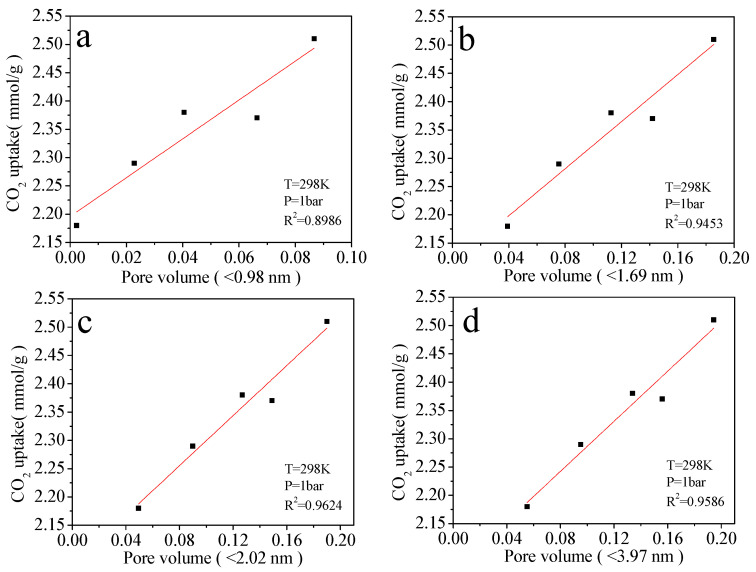
Linear fitting of CO_2_ uptake at 298 K with (**a**–**d**) pore volume for distinct pore sizes (0.98 nm, 1.69 nm, 2.02 nm, 3.97 nm).

Figure 6 presents the cumulative pore volume distribution as a function of pore width for the LPSP-T series, derived from non-local density functional theory (NLDFT) analysis of N_2_ adsorption–desorption isotherms. Among the samples, LPSP-700 exhibited the highest cumulative pore volume (~0.30 cm^3^/g), followed closely by LPSP-750 and LPSP-650, indicating that these materials possess a highly developed porous network. The pronounced increase in cumulative pore volume at pore widths below ~3 nm reflects the abundant presence of micropores and narrow mesopores, which are known to play a pivotal role in CO_2_ capture at near-ambient pressures. In addition, the gradual increase in pore volume extending to the mesopore region (2–35 nm) confirms the existence of hierarchical porosity, which benefits gas transport and facilitates the accessibility of active adsorption sites. The structure–adsorption property relationship was further examined by correlating pore size distribution with CO_2_ uptake, as presented in Figure 7. This figure illustrates the linear relationship between CO_2_ uptake at 25 °C and cumulative pore volume in selected pore size ranges for the LPSP-T series. The four subplots (a–d) correspond to pore widths of <0.98 nm, <1.69 nm, <2.02 nm, and <3.97 nm, respectively. In each case, a clear positive correlation was observed, confirming that the pore volume within these ranges significantly influences adsorption capacity. Notably, the most pronounced dependence of CO_2_ uptake on pore volume is observed for pores smaller than 2.02 nm (Figure 7c), where the coefficient of determination (R^2^) reaches as high as ~0.96. This provides compelling evidence that narrow micropores and micropores (<2 nm) are the dominant contributors to CO_2_ uptake under ambient conditions, owing to their strong confinement effects and enhanced adsorbate–adsorbent interactions. Collectively, these results demonstrate that CO_2_ adsorption in LPSP carbons is primarily governed by the availability of narrow micropores, while mesopores act in a complementary role by ensuring efficient diffusion pathways and minimizing mass-transfer resistance. Thus, the coexistence of micropores (responsible for adsorption capacity) and mesopores (responsible for transport kinetics) creates a synergistic hierarchical pore structure, which is crucial for achieving high-performance CO_2_ sorbents.

**Figure 8:**
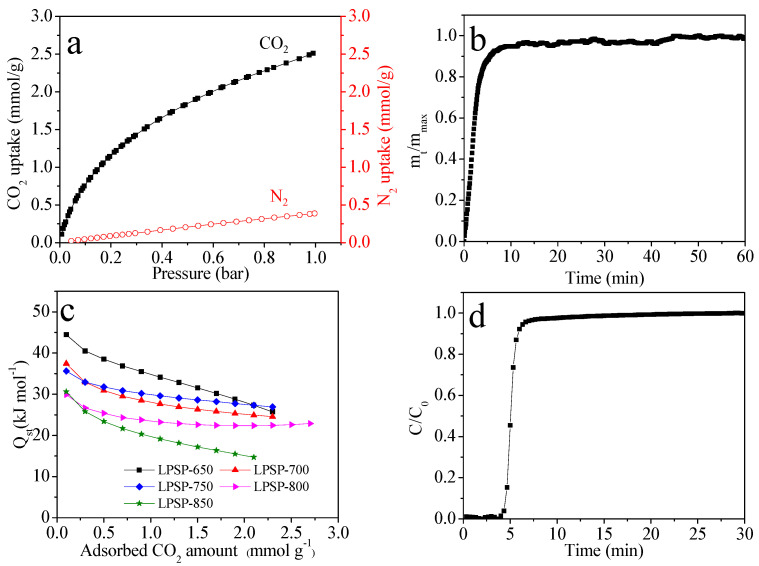
(**a**) CO_2_ and N_2_ isotherms of LPSP-700 at 25 °C and 1 bar, (**b**) adsorption kinetic of CO_2_ at 25 °C for LPSP-700, (**c**) *Q_st_* of CO_2_ adsorption on LPSP-T adsorbents derived from the experimental adsorption isotherms at 0 and 25 °C and (**d**) breakthrough plots of LPSP-700 (adsorption temperature: 25 °C, gas flow rate: 10 mL/min, inlet CO_2_ concentration: 10 vol.%, gas pressure: 1 bar).

Figure 8 comprehensively evaluates the CO_2_ capture performance of the LPSP series, with particular emphasis on LPSP-700 owing to its superior adsorption characteristics. As shown in Figure 8a, the CO_2_ and N_2_ adsorption isotherms at 25 °C up to 1 bar clearly demonstrate that LPSP-700 possesses a markedly stronger affinity for CO_2_ than for N_2_. At 1 bar, the CO_2_ uptake reaches ~2.6 mmol/g, while N_2_ uptake remains as low as ~0.25 mmol/g, underscoring the material’s excellent CO_2_ selectivity. Correspondingly, the calculated IAST [49] selectivity for a CO_2_/N_2_ mixture (10:90, *v*/*v*) is 19, confirming the strong potential of LPSP-700 for post-combustion CO_2_ separation.

Figure 8b illustrates the CO_2_ adsorption kinetics of LPSP-700 at 25 °C. Nearly 90% of the equilibrium uptake is achieved within 6 min, indicating rapid diffusion and efficient accessibility of active pores. Such fast adsorption kinetics are advantageous for practical operation, especially in cyclic adsorption–desorption processes under dynamic flow conditions.

The adsorption strength was further investigated by the isosteric heat of adsorption (*Q_st_*) (Figure 8c), derived from isotherms at 0 °C and 25 °C. At near-zero coverage, LPSP-700 exhibits the highest *Q_st_* of ~44 kJ/mol, gradually decreasing to ~15 kJ/mol with increasing loading. This decline reflects the progressive filling of adsorption sites with different binding energies. The overall moderate *Q_st_* values (15–44 kJ/mol) indicate that CO_2_ adsorption on LPSP-700 is predominantly physisorptive in nature: sufficiently strong for selective capture yet reversible, which is favorable for long-term cyclic operation.

Finally, Figure 8d depicts the dynamic breakthrough behavior of LPSP-700 under simulated post-combustion conditions (25 °C, 1 bar, 10 vol.% CO_2_, 10 mL/min total flow). The breakthrough curve exhibits a sharp and well-defined mass transfer zone, indicative of efficient adsorption dynamics. The calculated dynamic CO_2_ uptake is 0.81 mmol/g, closely matching the equilibrium capacity, thereby validating the stability and reliability of LPSP-700 under continuous flow operation.

**Figure 9:**
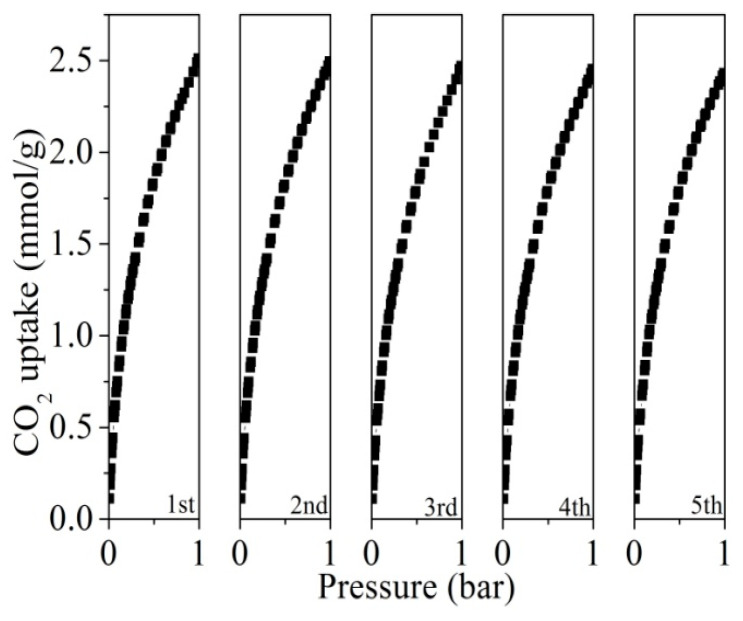
Cyclic study of CO_2_ adsorption for LPSP-700.

Building upon the equilibrium, kinetic, and breakthrough results in Figure 8, which collectively demonstrate the high capacity, fast diffusion, and robust dynamic performance of LPSP-700, Figure 9 further assesses its cyclic CO_2_ adsorption–desorption behavior to evaluate regeneration and long-term operational stability. Remarkably, the CO_2_ uptake capacity remains virtually unchanged over five consecutive cycles, with negligible performance loss. This outstanding recyclability highlights not only the structural robustness of LPSP-700 but also the high reversibility of the adsorption process, thereby reinforcing its practical potential for cyclic CO_2_ capture in real-world post-combustion scenarios.

## 4. Conclusions

In summary, phosphorus-doped porous carbons were successfully synthesized via a one-step phosphorus doping and thermal activation strategy using lotus petiole biomass and sodium phytate as the precursor and dual-function agent, respectively. The activation temperature was found to be a key parameter in regulating the surface area, pore structure, and heteroatom incorporation. Among the series, LPSP-700 exhibited the most favorable characteristics, with a high BET surface area of 525 m^2^/g, a total pore volume of 0.39 cm^3^/g, and a narrow micropore volume of 0.28 cm^3^/g. These structural advantages, together with P-containing functional groups, endowed LPSP-700 with an excellent CO_2_ uptake of 2.51 mmol/g at 25 °C and 3.34 mmol/g at 0 °C under 1 bar. Moreover, LPSP-700 demonstrated rapid adsorption kinetics (reaching 90% equilibrium capacity within 6 min), superior CO_2_/N_2_ selectivity (19 under 10:90 CO_2_/N_2_ mixture), and moderate isosteric heats of adsorption (15–44 kJ/mol), ensuring both high affinity and easy regeneration. Breakthrough experiments further confirmed its dynamic CO_2_ capture capacity (0.81 mmol/g), while cyclic tests revealed outstanding stability with negligible performance loss over at least five adsorption–desorption cycles. Importantly, micropore structure analysis highlighted the dominant role of micropores (<2.02 nm) in CO_2_ capture, as supported by the strong correlation (R^2^ ≈ 0.9624) between the uptake and micropore volume. Overall, this work demonstrates that the synergistic integration of optimized hierarchical porosity and phosphorus doping enables LPSP-700 to serve as a robust, sustainable, and high-performance sorbent for post-combustion CO_2_ capture.

## Figures and Tables

**Table 1:** Porous properties, elemental compositions, and CO_2_ uptakes of precursors and sorbents derived from different conditions.

Sample	S_BET_ ^a^ (m^2^/g)	V_0_ ^b^ (cm^3^/g)	V_t_ ^c^ (cm^3^/g)	V_n_ ^d^ (cm^3^/g)	XPS (at. %)				CO_2_ Uptake (mmol/g)	
					N	C	P	O	25 °C	0 °C
LPC	2	0.10	-	-	1.49	78.51	0.57	19.43	0.23	0.36
LPSP-650	418	0.31	0.15	0.25	1.49	73.25	2.84	22.42	2.37	3.07
LPSP-700	525	0.39	0.19	0.28	1.58	73.06	2.92	22.44	2.51	3.34
LPSP-750	341	0.32	0.12	0.26	1.40	71.88	3.28	23.43	2.38	3.17
LPSP-800	237	0.26	0.08	0.28	1.46	72.42	2.45	23.66	2.29	3.01
LPSP-850	132	0.19	0.04	0.28	1.31	72.11	3.09	23.49	2.18	2.63

^a^: Surface area was calculated using the BET method at P/P_0_ = 0.005-0.05. ^b^: Total pore volume at P/P_0_= 0.99. ^c^: Evaluated by the t-plot method. ^d^: Pore volume of narrow micropores (<1 nm) obtained from the CO_2_ adsorption data at 0°C.

## Data Availability

The data presented in this study are available on request from the corresponding author.

## Supplementary Materials

The following supporting information can be downloaded at: https://www.mdpi.com/article/10.3390/molecules30193990/s1, Scheme S1. Schematic diagram of the fixed-bed reactor system. Figure S1. Schematic diagram of the synthesis of lotus petiole derived P-doped porous carbons.
