# Supplementary material for: Novel Synthesis of Phosphorus-Doped Porous Carbons from Lotus Petiole Using Sodium Phytate for Selective CO2 Capture
Source: Molecules. 2025 Oct 5;30(19):3990. doi: 10.3390/molecules30193990 (PMC12526292; doi:10.3390/molecules30193990)
Supplement: Supplementary file 1 [file molecules-30-03990-s001.zip › molecules-3881664-supplementary.pdf]

Novel Synthesis of Phosphorus-Doped Porous Carbons from Lotus Petiole using Sodium Phytate for Selective CO_2 Capture

(Supplementary Materials)

Sodium phytate activation

For a typical reaction, 2 g LPC was combined with a solution that contained 4g sodium phytate. After stirring vigorously for 6 h, the mixture was left overnight to dry at 120 °C in an oven. Afterwards, the sample was activated to 700 °C for 2 h. During the activation process, the heating rate is 5 °C/min and nitrogen flow rate is 400 mL/min. Following activation, the sorbent was rinsed with distilled water until the pH value of the filtrate was roughly 7. The wet sample was then dried at 150 °C under vacuum for 24 h. The obtained sample was denoted as LPSP-700.

Characterization

Powdered X-ray diffraction (XRD) patterns were carried out on a PHILIPS PW3040/60 powder diffractometer using CuKα radiation (λ =0.15406nm). Scanning electron microscopy (SEM Hitachi S-4800) was used to observe the morphology of the samples of carbon materials. Further details of the pore structure were determined by transmission electron microscopy (TEM, JEOL-2100F) operated at 200 kV. Nitrogen adsorption and desorption isotherms were measured on a Beishide 3H-2000PS2 sorption analyzer at -196ºC. Ultrahigh-purity N_2 (99.999%, Shanghai Pujiang Gas Co., Ltd) was used for measurement. Before measurement, the samples were degassed in a vacuum at 200ºC for at least 12h. The specific surface area (S_{BET}) was calculated according to the multipoint Brunauer-Emmett-Teller (BET) method from the adsorption data in the relative pressure range between 0.005 and 0.05. The total micropore volume (V_t) was deduced from the N_2 adsorption

data by the t-plot method, and the total pore volume (V_0) was estimated from the adsorbed amount of liquid nitrogen at a relative pressure of 0.99. The error of porosity measurement is within 3%. The pore size distribution was calculated using the density functional theory (DFT) method. In addition, X-ray photoelectron (XPS) measurements were performed using an AXIS Nova spectrometer (Kratos Inc., NY, USA) equipped with a monochromatic Al Kα X-ray source (1486.6 eV). XPS survey spectra were recorded with a pass energy of 160 eV, and high-resolution spectra with a pass energy of 40 eV.

The CO_2 adsorption isotherms were measured using the Beshide 3H-2000PS2 sorption analyzer at 0ºC and 25ºC, respectively. Pure CO_2 (99.99%, Shanghai Pujiang Gas Co., Ltd) was used for adsorption. Prior to each adsorption experiment, the sample was degassed for 12 h at 200ºC to remove the guest molecules from the pores. The volume of narrow micropores (with sizes <1 nm), V_n, was calculated from CO_2 adsorption at 0°C using the Dubinin–Radushkevich (D-R) equation. The measurements were repeated for each sample, until the values fell within ± 2% of each other.

Measurement of dynamic CO_2 uptake of the sorbents

The dynamic CO_2 uptake of the sorbents was tested on a fixed-bed reactor schematically illustrated in Scheme S1 at 1 bar and 25 °C. First, the sample was heated at 100°C for 1 h under N_2 at a flow rate of 20 mL/min. The gas flow was shifted from nitrogen to a 10% mixture of CO_2 in N_2 at a flow rate of 10 mL/min, when the sample temperature was lowered to 25°C. The effluent gases were monitored online using an Agilent 7820A gas chromatograph with a thermal conductivity detector (TCD). From the breakthrough curves, the dynamic CO_2 capture capacity on an adsorbent was calculated. The error for this measurement falls within 2%.

Measurement of CO_2 adsorption kinetics

The adsorption kinetics of CO_2 was measured in a thermogravimetric analyzer (NETZSCH STA 449C). In the kinetic analysis, the sample (~5 mg) was degassed under a He stream at 200°C for 1 h. Next, the temperature was cooled to the experimental temperature of 25°C. Then the CO_2 gas was fed into the test chamber with a flow rate of 50 mL/min and the weight variation with time was recorded. The measurement' s error is within 2%.

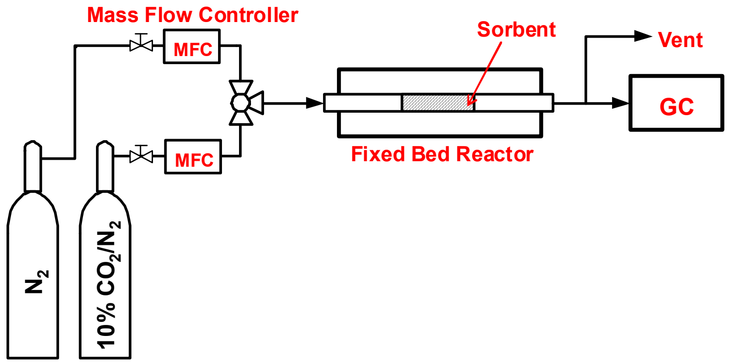

Scheme S1. Schematic of the fixed-bed reactor system.

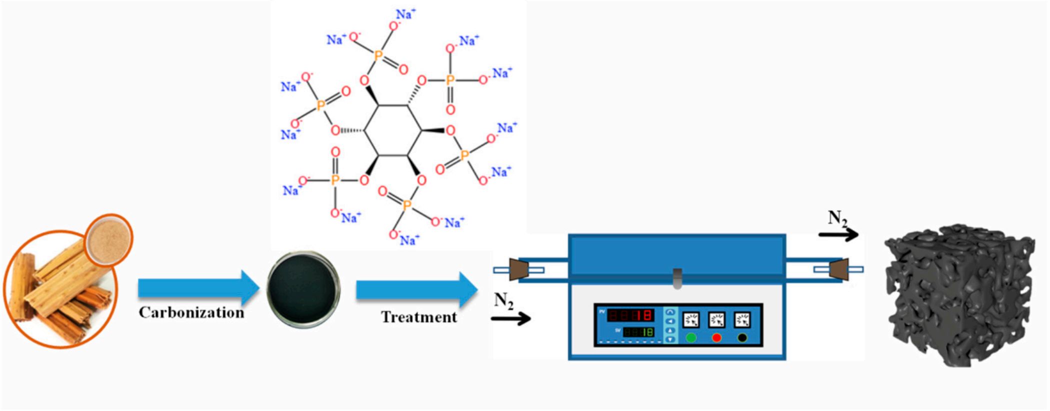

Figure S1. Schematic diagram of the synthesis of lotus petiole derived P-doped porous carbons.
